# Neurologic deficits due to proximal junctional kyphosis after adult spinal deformity surgery: how often do they happen and do they improve?

**DOI:** 10.1007/s43390-026-01309-x

**Published:** 2026-02-18

**Authors:** Hani Chanbour, Alan R. Tang, Harsh Jain, Alexander T. Lyons, Soren Jonzzon, Iyan Younus, Steven G. Roth, Amir M. Abtahi, Byron F. Stephens, Scott L. Zuckerman

**Affiliations:** 1 Department of Neurological Surgery, Vanderbilt University Medical Center, Medical Center North T-4224, Nashville, TN 37212 USA; 2 School of Medicine, Vanderbilt University, Nashville, TN USA; 3 Department of Orthopedic Surgery, Vanderbilt University Medical Center, Nashville, TN USA

**Keywords:** Adult spinal deformity, Neurological deficits, Proximal junctional failure, Proximal junctional kyphosis

## Abstract

**Purpose:**

Rate of neurological deficits in patients with PJK/F after adult spinal deformity (ASD) surgery is understudied. In patients undergoing reoperation for proximal junctional kyphosis/failure (PJK/F) after adult spinal deformity (ASD) surgery, we sought to: 1) report the rate of neurological deficit, 2) describe these deficits, and 3) discuss improvement rates after reoperation.

**Methods:**

A retrospective cohort study (2009–21) included ASD patients with: ≥ five-level fusion, sagittal/coronal deformity, and > 2-year follow-up. PJK was defined as 10º Cobb angle of the upper instrumented vertebra (UIV)–UIV + 2, and 10º increase from preoperative, while PJF was catastrophic PJK requiring reoperation due to screw pullout/fracture. Primary outcome was the presence of neurological deficits and/or worsened motor exam at the time of PJK/F diagnosis, description of deficits, and improvement after reoperation.

**Results:**

Of 238 ASD patients, 47(19.7%) underwent reoperation for PJK/F (mean age: 69.6 ± 9.4 years; mean instrumented levels: 9.4 ± 2.7) at a median of 15.6 months (IQR: 9.8–25.5). New motor deficits were seen in 15/47(31.9%) patients at the time of PJK/F diagnosis, and 3 (6.4%) endorsed incontinence. Neurological deficits occurred in 8/29 (24.1%) PJK and 7/18 (44.4%) PJF patients (*p* = 0.147). Distal motor strength was 0/5 in 1 (6.7%), 3/5 in 5 (33.3%), and 4/5 in 9 (60.0%). Of 15 patients with new motor deficits, 5 (33%) immediately returned-to-baseline, 5 (33%) improved but not to baseline, 3 (20%) remained unchanged, and 2 (13%) worsened within 3 months of reoperation. At the most recent follow-up (median: 3.9 years), 13/15 (86.7%) patients had no deficits, and 2 (13.3%) improved but not to baseline. Notably, 1 patient without neurological deficit preoperatively developed 4/5 motor exam following PJK surgery.

**Conclusions:**

Among patients reoperated for PJK/F, 32% had motor deficits and 6% had bladder incontinence. Immediately post-surgery, 33% returned-to-baseline, at last follow-up, 87% returned-to-baseline. These results can help patients, families, and surgeons appreciate long-term neurologic function after reoperation for PJK/F.

## Introduction

Symptomatic adult spinal deformity (ASD) is an increasingly significant source of disability worldwide, with rates of surgical correction increasing in patients refractory to nonoperative treatment [1, 2]. Surgical intervention aims to decompress neural elements, improve sagittal/coronal alignment, and alleviate pain; however, ASD surgery can lead to high rates of mechanical complications [3]. In particular, proximal junctional kyphosis (PJK) and proximal junctional failure (PJF) are well-recognized and vexing complications following long-segment instrumented spinal fusion for ASD [4–7]. While PJK can be limited to a focal kyphosis at the upper instrumented vertebra (UIV), PJF occurs as a catastrophic structural failure defined by vertebral body fracture or screw pullout, often requiring reoperation [8–12].

Symptomatic PJK/F can require reoperation, and in severe cases, lead to spinal cord compression with a neurologic deficit [13–16]. The incidence of neurologic deficit due to PJK/F has been reported between 0 and 27% [17–19], but the severity, specific deficits, and recovery remain unknown. In a retrospective study of 385 operative ASD patients, PJF with late neurological deficits developed in 4.7% of patients, and patients undergoing subsequent revision surgery were noted to have high perioperative complication rates and poor prognosis for improved neurological status [17]. Studies devoted to the rate, reason, and outcomes of neurologic deficits are lacking.

Identifying the rate and specific nature of neurological deficits in patients with PJK/F is critical in developing appropriate management strategies to minimize their occurrence and improve neurological outcomes. Moreover, explaining the incidence and prognosis of catastrophic neurologic deficits to patients is important in the decision to undergo surgery. Therefore, in a cohort of patients undergoing ASD surgery, we sought to: 1) report the rate of neurological deficit in patients undergoing reoperation for PJK/F, 2) describe these deficits, and 3) discuss the rate of improvement after reoperation.

## Methods

### Study design

A retrospective, cohort study was conducted at a single institution from 2009 to 2021 of patients undergoing ASD surgery with at least a 2-year follow-up. All ASD surgeries were performed by five fellowship-trained neurosurgery and orthopedic spine surgeons. Institutional Review Board (IRB) approval was obtained (IRB #220894). Informed consent was waived due to the retrospective nature of the study.

### Patient population

Inclusion criteria for ASD surgery were defined as adults age ≥ 18 years undergoing elective ≥ five-level fusions, Cobb angle ≥ 30°, sagittal vertical axis (SVA) ≥ 5 cm, coronal vertical axis (CVA) ≥ 3 cm, pelvic tilt (PT) of ≥ 25°, or thoracic kyphosis (TK) ≥ 60°. All patients included in the study had a minimum of 2-year follow-up.

### Exposure variable

The primary exposure variable of interest was reoperation for PJK/F. PJK was identified and defined radiographically as an angle between the inferior endplate of the UIV and the superior endplate of the UIV + 2 vertebra ≥ 10° with a concomitant ≥ 10° change compared to preoperative imaging, consistent with prior studies [7, 14, 20]. While the threshold of the kyphotic angle may be low, this definition of PJK by Glattes et al. [20] is the most widely accepted in the literature. PJF was defined as PJK with structural compromise, including vertebral body fracture, posterior ligament complex, and vertebral subluxation or need for reoperation [20, 21]. The preoperative and postoperative alignment, after the index surgery, was described using pelvic incidence (PI), PT, SS, SVA, and L1-pelvic angle (L1PA). Nadler’s formula was used to calculate the estimated blood volume [14].

### Neurological deficit

The primary outcome variable was the presence of neurological deficits, defined as a worsened motor/sensory exam at the time of PJK/F diagnosis. Motor strength score and American Spinal Injury Association (ASIA) Impairment Scale were reported [22]. For motor strength, six-point ordinal scoring, Medical Research Council (MRC) grading scales were used: Grade 0: no contraction; Grade 1: visible or palpable contraction, but no movement; Grade 2: movement with gravity eliminated; Grade 3: movement against gravity, but not against resistance; Grade 4: movement against resistance, but with weakness; and Grade 5: normal strength. Improvement and deterioration were defined as an increase or reduction in scores postoperatively. Bladder and/or bowel incontinence were also reported [23–25].

Secondary outcomes included a change in neurological deficits or motor exam following reoperation for PJK/F. Patient-reported outcome metrics (PROMs), including Oswestry Disability Index (ODI), Numeric Rating Scale for back pain (NRS–BP) and leg pain (NRS–LP), and EuroQol 5D (EQ5D) at 2 years, were also reported. Due to the importance of long-term follow-up to monitor neurologic deficits, patients were called via telephone to gain a detailed assessment of their neurologic status.

### Statistical analysis

Descriptive statistics were used to compare PJK/F patients undergoing reoperation with and without neurological deficits. Continuous variables were reported as means and standard deviations, while categorical variables were reported as frequencies. The Shapiro–Wilk test and *F* test were employed to evaluate normal distribution and variance for continuous variables, respectively. Normally distributed data with equal variance were analyzed using a two-tailed *t* test, while nonparametric data were compared with the Wilcoxon signed-rank or Mann–Whitney test. For nominal data, χ2 or Fisher’s exact test was used in smaller samples with smaller frequencies. Receiver-operating characteristic (ROC) curve analysis was performed to determine whether proximal junctional angle correlates with occurrence of neurological deficits. A significance level of *p* value < 0.05 was considered statistically significant. All analyses were performed using R version 4.2.1 (R Foundation, Vienna, Austria).

## Results

### Patient demographics and radiographic alignment

**Fig. 1:**
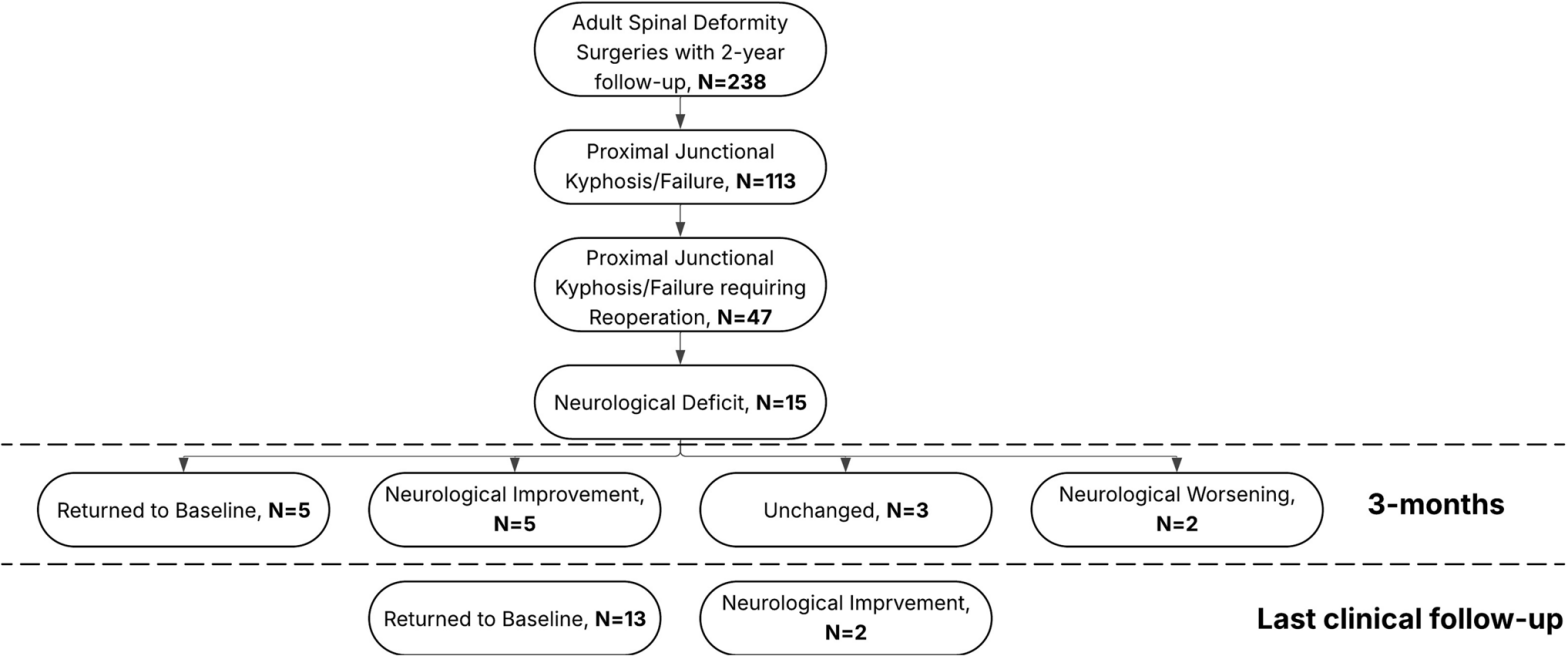
Flowchart for the patient population in the study

Of 238 ASD patients, 47 (19.7%) underwent reoperation for PJK/F at a median (IQR) of 15.6 months (9.8–25.5) (Fig. 1). Out of these reoperation patients, 29 were diagnosed with PJK (61.7%), and 18 (38.3%) were diagnosed with PJF, according to previously published criteria [20]. Compared to those without neurological deficits (*n* = 32), patients with deficits (*n* = 15) were of similar age (70.9 ± 8.1 vs. 69.0 ± 10.1 years, p = 0.544), sex (13.3% vs. 31.3% male, *p* = 0.288), and BMI (29.6 ± 6.3 vs. 29.7 ± 5.4, *p* = 0.933). Rates of ≥ 2 comorbidities (73.3% vs. 53.1%, *p* = 0.251), prior fusion (40.0% vs. 34.4%, *p* = 0.708), and smoking history (*p* = 0.954) were also similar.

Preoperative radiographic alignment and PROMs, including ODI, NRS–BP, NRS–LP, and EQ5D, were comparable between the groups. Surgical factors, including total instrumented levels (9.6 ± 2.3 vs. 9.3 ± 2.9, *p* = 0.767), mean blood loss as a proportion of blood volume (41.8 ± 31.2% vs. 39.8 ± 34.0%, *p* = 0.847), operative time (445.1 ± 216.9 vs. 423.8 ± 147.3 min, *p* = 0.693), and rate of home discharge (*p* = 1.000) were also similar. Population characteristics are summarized in Table 1.

**Table 1:** Demographic and index surgery variables

Variable	Total, *N* = 47	With neurological deficits, *N* = 15	Without neurological deficits, *N* = 32	*p* value
Age (years), mean ± SD	69.6 ± 9.5	70.9 ± 8.1	69.0 ± 10.1	0.544
Sex, male, n (%)	12 (25.5%)	2 (13.3%)	10 (31.3%)	0.288
BMI, mean ± SD	29.7 ± 5.6	29.6 ± 6.3	29.7 ± 5.4	0.933
Comorbidities, n (%)				0.251
0	4 (8.5%)	0 (0.0%)	4 (12.5%)	
1	15 (31.9%)	4 (26.7%)	11 (34.4%)	
2 +	28 (59.6%)	11 (73.3%)	17 (53.1%)	
Smoking status				0.954
Current	3 (6.5%)	1 (6.7%)	2 (6.5%)	
Prior	14 (30.4%)	5 (33.3%)	9 (29.0%)	
Never	29 (63.0%)	9 (60.0%)	20 (64.5%)	
Osteoporosis, n (%)	7 (14.9%)	2 (13.3%)	5 (15.6%)	1.000
T-score	− 1.5 ± 1.1	− 1.6 ± 1.2	− 1.4 ± 1.0	0.749
Diabetes, n (%)	11 (23.4%)	5 (33.3%)	6 (18.8%)	0.271
COPD, n (%)	19 (40.4%)	8 (53.3%)	11 (34.4%)	0.217
Heart failure, n (%)	9 (19.1%)	4 (26.7%)	5 (15.6%)	0.438
Hypertension, n (%)	41 (87.2%)	14 (93.3%)	27 (84.4%)	0.648
Prior fusion, (%)	17 (36.2%)	6 (40.0%)	11 (34.4%)	0.708
Preoperative radiographic alignment, mean ± SD				
Pelvic incidence (°)	52.9± 26.6	53.2 ± 16.2	52.7 ± 13.5	0.916
Pelvic tilt (°)	26.6 ± 10.8	27.5 ± 12.5	26.2 ± 10.1	0.725
Sacral slope (°)	26.6 ± 11.1	26.9 ± 11.2	26.4 ± 11.2	0.897
Sagittal vertical axis (mm)	87.0± 62.3	90.5 ± 68.9	85.7 ± 70.5	0.840
L4–S1 lordosis (°)	− 31.4 ± 16.9	− 28.3 ± 18.9	− 32.8 ± 16.3	0.499
L1PA (°)	14.4 ± 11.3	17.5 ± 16.8	13.2 ± 8.9	0.363
L1PA within 3° of target, n (%)	6 (20.0%)	0 (0.0%)	6 (27.3%)	0.155
Preoperative PROMs, mean ± SD				
ODI	53.8 ± 11.5	56.2 ± 10.9	52.2 ± 11.8	0.316
NRS–BP	7.3 ± 1.7	7.2 ± 1.7	7.2 ± 1.7	0.778
NRS–LP	6.9 ± 2.0	6.6 ± 1.3	7.2 ± 2.3	0.370
EQ5D	0.5 ± 0.2	0.4 ± 0.2	0.5 ± 0.3	0.159
Index surgery				
Pedicle subtraction osteotomy, n (%)	7 (14.9%)	2 (13.3%)	5 (15.6%)	1.000
Three-column osteotomy, n (%)	7 (14.9%)	2 (13.3%)	5 (15.6%)	1.000
Total instrumented levels, mean ± SD	9.4 ± 2.7	9.6 ± 2.3	9.3 ± 2.9	0.767
Estimated blood volume (ml), mean ± SD	4245.2 ± 810.8	4009.6 ± 952.9	4355.7 ± 725.2	0.175
Estimated blood loss (ml), mean ± SD	1690.7 ± 146,728	1629.0 ± 1170.0	1719.7 ± 1604.2	0.846
Blood loss/blood volume (%), mean ± SD	40.5 ± 32.8	41.8 ± 31.2	39.8 ± 34.0	0.847
Operative time (min), mean ± SD	463.1 ± 158.7	445.1 ± 216.9	423.8 ± 147.3	0.693
Length of stay (days), mean ± SD	6.7 ± 3.8	6.3 ± 2.3	6.9 ± 4.4	0.619
Discharge home, n (%)	22 (50.0%)	7 (50.0%)	15 (50.0%)	1.000
Proximal junctional kyphosis, n (%)	29 (61.7%)	8 (53.3%)	21 (65.6%)	0.419
Proximal junctional fracture, n (%)	18 (38.3%)	7 (46.7%)	11 (34.4%)	0.419
Postoperative radiographic alignment, mean ± SD				
Pelvic incidence (°)	53.7 ± 13.5	55.8 ± 12.6	52.4 ± 14.2	0.559
Pelvic tilt (°)	26.2 ± 9.0	27.7 ± 11.2	25.5 ± 7.7	0.436
Sacral slope (°)	26.1 ± 9.0	25.6 ± 11.4	26.4 ± 7.5	0.797
Sagittal vertical axis (mm)	60.8 ± 60.9	61.1 ± 49.9	60.6 ± 66.6	0.982
L4–S1 lordosis (°)	−32.4 ± 10.0	−28.4 ± 10.8	−34.9 ± 8.9	0.126
L1PA (°)	11.8 ± 9.5	16.1 ± 10.1	9.8 ± 8.8	0.148
L1PA within 3° of target, n (%)	5 (25.0%)	0 (0.0%)	5 (38.5%)	0.114
PROMs—2 years, mean ± SD				
ODI	46.3 ± 20.3	49.4 ± 18.5	44.6 ± 21.5	0.520
NRS–BP	5.6 ± 2.8	6.3 ± 2.7	5.1 ± 2.7	0.231
NRS–LP	3.6 ± 3.7	4.3 ± 3.0	3.2 ± 3.6	0.385
EQ5D	0.6 ± 0.2	0.5 ± 0.3	0.6 ± 0.2	0.228

Postoperative radiographic alignment parameters were similar between patients with and without neurological deficits. PI (55.8 ± 12.6° vs. 52.4 ± 14.2°, *p* = 0.559), PT (27.7 ± 11.2° vs. 25.5 ± 7.7°, *p* = 0.436), and SS (25.6 ± 11.4° vs. 26.4 ± 7.5°, *p* = 0.797) were comparable across groups. Similarly, no significant differences were observed in SVA (61.1 ± 49.9 vs. 60.6 ± 66.6 mm, *p* = 0.982), L4–S1 lordosis (–28.4 ± 10.8° vs. –34.9 ± 8.9°, *p* = 0.126), or L1PA (16.1 ± 10.1° vs. 9.8 ± 8.8°, *p* = 0.148). None (0.0%) of the patients with neurological deficits had L1PA within the target, compared to 38.5% of those without neurological deficits, although this was not statistically significant (*p* = 0.114) (Table 1).

### Neurological deficit

Of patients undergoing reoperation for PJK/PJF (*n* = 47), 15/47 (31.9%) patients exhibited new motor deficits at the time of PJK/F diagnosis, and 3 (6.4%) patients had incontinence. Of PJK patients, 8/29 (24.1%) exhibited neurological deficits, while 7/18 (44.4%) PJF patients exhibited neurological deficits (*p* = 0.147).

### Description of neurological deficit

Of 15/47 patients with neurological deficit, ASIA grade was A (N = 0, 0%), B (N = 1, 7.0%), C (N = 1, 7.0%), D (N = 13, 86.0%), and E (N = 0, 0%). Distal motor strength was 0/5 in 1 (6.6%) patient, 3/5 in 5 (33.4%) patients, and 4/5 in 9 (60.0%) patients. The mean time from symptom onset to reoperation was 178.2 ± 324.4 days.

### Risk factors for PJF and PJK in patients with neurologic deficits

Among the patients with neurologic deficits, seven (46.7%) patients had PJF, while eight (53.3%) had PJK. Compared to patients who developed PJK and neurological deficits, those who developed PJF with neurological deficits were more likely to have had a prior fusion surgery before their index ASD surgery (71.4% vs. 12.5%, *p* = 0.041). There was no significant difference in age (70.1 ± 11.1 vs. 71.7 ± 3.0 years, *p* = 0.709), BMI (28.5 ± 6.5 vs. 30.7 ± 6.5, *p* = 0.522), comorbidities (*p* > 0.999), osteoporosis (0.0% vs. 25.0%, *p* = 0.467) or change in L1–S1 lordosis (−6.4 ± 14.0 vs. 2.6 ± 28.1, *p* = 0.469) between patients with PJK vs. PJF who also developed neurological deficits.

### Proximal junctional angle threshold for neurological deficits

In the patients with PJF, the preoperative proximal junctional angle was 30.1 ± 20.3° in those with neurological deficits, while it was 24.5 ± 9.5° in those without deficits, with no significant difference (*p* = 0.630). This difference was also not statistically significant in patients with PJK (24.6 ± 15.0° vs. 22.3 ± 11.6°, *p* = 0.720) or with either PJK or PJF (27.3 ± 17.1° vs. 23.1 ± 10.8°, *p* = 0.408). ROC analysis was done to assess the performance of the proximal junctional angle as a predictor of neurological deficits. The proximal junctional angle in patients with PJF (AUC = 0.53, 95% CI = 0.14–0.92, *p* = 0.867), PJK (AUC = 0.58, 95% CI = 0.25–0.90, *p* = 0.647) or PJK and/or PJF (AUC = 0.58, 95% CI = 0.34–0.81, *p* = 0.518), did not accurately predict neurological deficits. Due to low AUC values, Youden’s index was not subsequently calculated.

### Improvement of neurological deficits

Within 3 months of the reoperation, 5 (33%) patients immediately returned to baseline, 5 (33%) had improved neurological deficit but did not return to baseline, 3 (20%) remained unchanged, and 2 (13%) worsened. Urinary incontinence was reported by 1/15 (6.7%) patients preoperatively, which showed gradual improvement postoperatively. At the most recent clinical follow-up (median 3.9 years), 13 (87%) patients returned to baseline, and 2 patients improved but not to baseline (one patient had left lower extremity strength of 3/5, which improved to 4/5 at 754 days, and one patient had left lower extremity strength of 1/5, which improved to 3 + /5 at 1399 days). For patients who returned to baseline, median time to return to baseline was 194 days. Of note, 1 patient without neurological deficit preoperatively developed 4/5 motor exam in the bilateral lower extremity following PJK surgery, her hospital course was complicated by a myocardial infarction, requiring emergent intervention and her weakness was thought to be due to the cardiac insult rather than a spinal cord injury. Table 2 summarizes motor strength at the 3-month and last clinical follow-ups.

**Table 2:** Minimum postoperative power for patients with neurological deficits at the time of PJK/F diagnosis at the 3-month and last clinical follow-ups

Patient	Preoperative neurological exam	3-Month follow-up, *N* = 15	Last clinical follow-up, *N* = 15
1	LL 4 + /5	Intact	Intact
2	LL 4/5	Intact	Intact
3	RU 3/5	LL 1/5	LL 3 + /5
	LU 3/5		
	RL 3/5		
	LL 3/5		
4	LL 3/5	RU 4/5	Intact
		LU 4/5	
		RL 4/5	
		LL 4/5	
5	RL 4/5	Intact	Intact
	LL 4/5		
6	RL 4/5	RL 4/5	Intact
	LL 4/5	LL 4/5	
7	RL 3/5	RL 4/5	Intact
	LL 3/5	LL 4/5	
8	RL 0/5	RLE 1/5	Intact
9	RL 3/5	RL 2/5	Bladder Dysfunction +
	LL 3/5	LL 4/5	
	Bladder	Bladder	
	Dysfunction + + +	Dysfunction + +	
10	LU 4/5	BUE 4/5	Intact
11	RL 4/5	Intact	Intact
	LL 4/5		
12	RU 4/5	RL 4/5	Intact
	LU 4/5		
	RL 4/5		
	LL 4/5		
13	LU 4/5	LU 4 + /5	Intact
	RL 4/5		
14	RL 3/5	LL 4/5	LL 4/5
	LL 3/5		
15	RU 4/5	Intact	Intact
	LU 4/5		
	RL 4/5		
	LL 4/5		
Motor deficit	15 (100.0%)	10 (66.7%)	2 (13.3%)
Bladder dysfunction	1 (6.7%)	1 (6.7%)	1 (6.7%)

Long-term follow-up was obtained through phone calls with a mean follow-up of 11.1 ± 2.2 years. Of these 15 patients, 4 (26.7%) were deceased, and 7 (46.7%) did not respond to phone calls. Among the remaining four patients, one who had preoperative urinary incontinence reported significant improvement and was now continent. The other three patients (20.0%) reported difficulty with ambulation and required a rollator. Of these, two had been neurologically intact postoperatively but developed mobility difficulties several years after surgery, which they attributed to aging.

### Patient-reported outcome measures

At 2-year follow-up, PROMs remained similar between groups: ODI (49.4 ± 18.5 vs. 44.6 ± 21.5, *p* = 0.520), NRS–back pain (6.3 ± 2.7 vs. 5.1 ± 2.7, *p* = 0.231), NRS–leg pain (4.3 ± 3.0 vs. 3.2 ± 3.6, *p* = 0.385), and EQ-5D (0.5 ± 0.3 vs. 0.6 ± 0.2, *p* = 0.228) (Table 1).

**Fig. 2:**
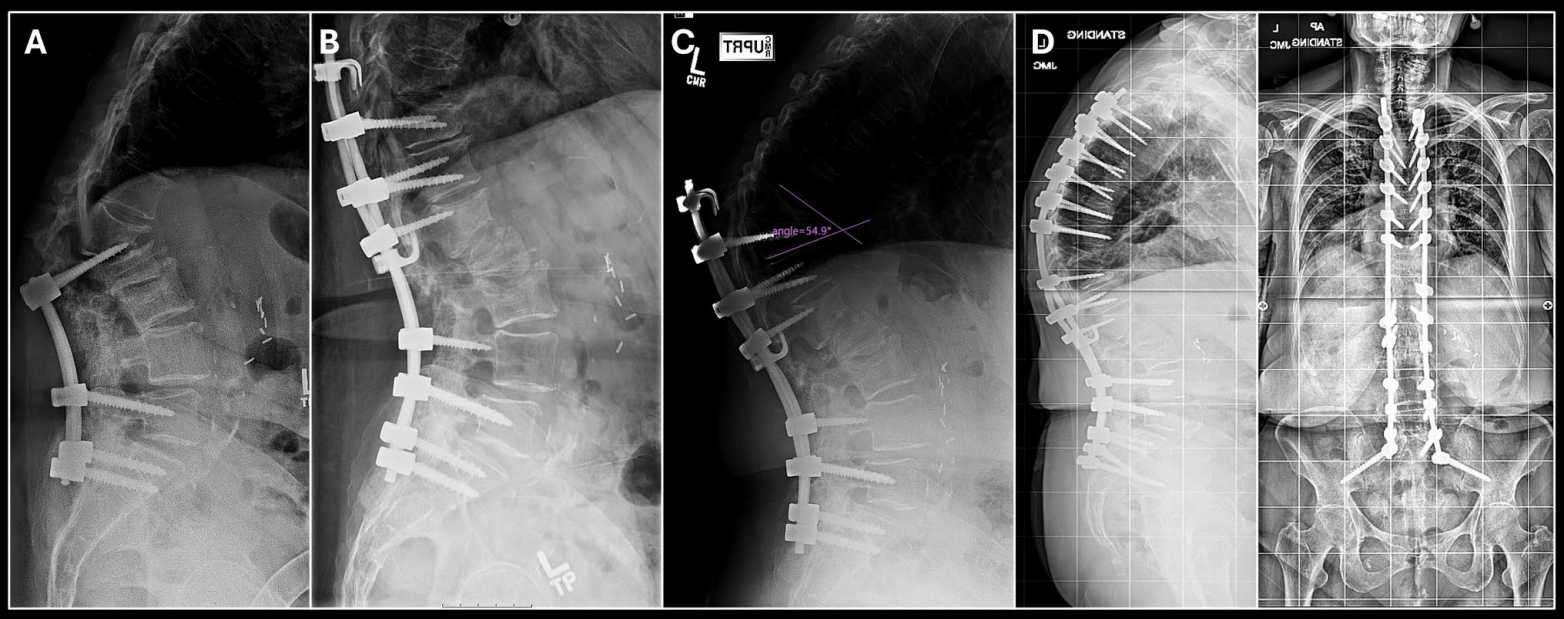
A 74-year-old female with a history of L2–S1 posterior lumbar fusion presented with acute low back pain, and inability to walk. **A** Preoperative lateral X-ray demonstrated L2 pedicle screw pullout and kyphotic deformity above the fusion construct due to a fracture dislocation at L1/2. **B** Patient underwent revision T12–L3 laminectomies and L2 transpedicular decompression with open reduction of kyphotic deformity and T10–S1 posterolateral lumbar fusion with postoperative lateral X-ray shown. **C** Three months postoperatively, the patient presented with worsening pain, kyphosis and a power of 3/5 with lateral X-ray showing new acute thoracic kyphosis. **D** Patient underwent revision T4–ilium posterolateral arthrodesis and instrumentation. Postoperative lateral and posterior–anterior X-rays are shown. Postoperatively, the power was 1/5 and improved to 4/5 at the last clinical follow-up

**Fig. 3:**
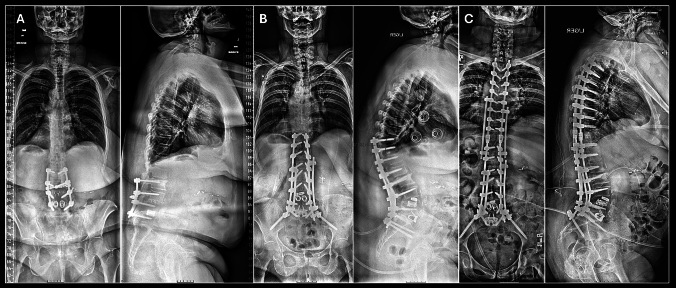
A 65-year-old obese female, with a history of smoking and prior L2–5 posterior spinal fusion, presented with chronic back and lower extremity pain. On examination, she had generalized weakness with 4 + /5 power in all muscle groups of the lower extremity. **A** Preoperative X-ray showed straightening of the thoracic kyphosis and lumbar lordosis, grade 1 anterolisthesis of L3 on L4 with multilevel degenerative changes and intervertebral disc space narrowing throughout the spine. The patient underwent L5–S1 PCOs, L4 PSO, T10–pelvis posterior spinal fusion, and L3–L5 anterior spinal fusion. Postoperatively, she had a power of 4/5 for dorsiflexion and plantar flexion bilaterally. **B** On postoperative day 5, X-rays showed a fracture at her T10 level and a kyphosis of 24°. (C) The patient underwent T9–10 laminectomy and decompression with an extension of fusion from T2-pelvis with postoperative X-rays shown. She had a power of 0/5 in her bilateral lower extremities postoperatively, which improved to 2–3/5 at discharge

Two representative cases with neurological improvement postoperatively are shown in Figs. 2 and 3.

## Discussion

While PJK/F is a vexing complication after ASD surgery, it becomes catastrophic if accompanied by a neurologic deficit. In patients undergoing reoperation for PJK/F, we evaluated the incidence, nature, and recovery of neurological deficits. Among 47 patients who underwent reoperation for PJK/F over the allotted time period, neurological deficits were observed in 32% of patients at the time of diagnosis of PJK/F, with a higher incidence in PJF (44%) compared to those with PJK (24%), though not statistically significant. Among patients with neurological deficits, those with PJF were significantly more likely to have had prior fusions before their deformity correction. Immediately after surgery, 33% returned to baseline, and at last follow-up, 87% returned to baseline. Long-term ambulatory issues were reported by 6% of the patients.

Among patients who underwent reoperation for PJK/F, approximately 1 in 3 (32%) had neurological deficits at the time of PJK/F diagnosis. The mechanism of these deficits is not surprising and is due to increased mechanical stress, structural deformities, or adjacent segment degeneration which irritate or compress neural structures [12, 26–28]. Several studies have reported the incidence of neurological deficits in PJF, suggesting an incidence of up to 27% [17–19]. In a study by Yagi et al. [19], 26% of the patients with PJF developed neurological deficits, and of these, 67% of the patients had coexisting spondylolisthesis. The incidence was as low as 5.5% in a study by Ha et al. [17] The reported rates of neurological deficits vary significantly depending on the population demographics and the follow-up period. Moreover, the decision of when to operate is surgeon-dependent, and surgeons who wait longer to operate on PJK/F may be more likely to have a neurologic deficit. We believe it is important to deal with PJK early, and at a minimum, follow patients with radiographic PJK very closely. While reoperating for PJK/F is unfortunate, adding neurologic compromise turns this complication into a potentially life-altering complication.

In an effort to predict who may develop neurologic deficits after PJK/F, the only significant predictor of a neurologic deficit was prior fusion surgery before the deformity correction. Reasons for this may have to do with spinal alignment. It is possible that patients with prior fusions may be fused flat, with suboptimal alignment, and unless a three-column osteotomy was performed, the malalignment may predispose to more severe PJK/F. The concept is supported by our observation that none of the patients with neurological deficits achieved an L1PA within the target range, suggesting that inadequate sagittal correction may contribute to increased mechanical stress at the proximal junction and subsequent neurological compromise. Of note, vertebropelvic angles and T4–L1–hip axis had not been published at the time most of our sample had surgery, which may have contributed to the overall lack of attaining specific alignment goals [29]. Moreover, prior fusions increase stress on the adjacent spinal segments, causing weakening of the bone, ligament and muscular structures, contributing to higher risks of developing neurological deficits in PJK/F. In contrast to our findings, prior studies have reported osteoporosis as a risk factor for neurological deficits in PJK/F [8], which was not seen in our data (13% vs. 16%). In addition, PJF with associated spondylolisthesis were more likely to have neurological deficits than those without spondylolisthesis, though no significant differences were noted in preoperative risk factors between these groups [19]. Although the literature studying risk factors for PJK/F is extensive, additional research is needed to elucidate predictors of neurologic deficits.

The finding that prior fusion was a significant risk factor among patients with neurological deficits and PJF has important implications for surgical planning. Patients with prior fusion often present with rigid, flat, or sub-optimally aligned constructs, which may limit the ability to achieve adequate sagittal correction with standard techniques [30, 31]. In this setting, more aggressive alignment strategies—including consideration of three-column osteotomies—may be necessary to restore appropriate global and regional alignment and reduce proximal junctional stress [30, 31]. In addition, careful selection of alignment targets, including parameters, such as L1PA, as well as thoughtful proximal construct selection, may be particularly important in this higher risk population to mitigate the risk of catastrophic junctional failure with associated neurological compromise. Moreover, spinal tethering may represent a strategy to reduce catastrophic failures at the UIV. An encouraging trend of neurological recovery was seen following reoperation of PJK/F with 67% of patients exhibiting at least some improvement 3 months postoperatively, and 87% returning to baseline at long-term follow-up. The timing of symptom onset to reoperation demonstrated substantial heterogeneity, likely reflecting differences in clinical presentation, symptom recognition, referral patterns, and surgical decision-making. Delays in operative intervention undoubtedly lead to prolonged spinal cord compression, which could adversely affect early neurological recovery, whereas earlier intervention in patients with acute or progressive deficits may partially explain a more favorable short-term outcomes [17, 32]. This finding is in line with the existing literature, which although limited, shows neurological improvement till the last follow-up (mean: 4.3 years) postoperatively [8, 32]. However, Ha et al. [17] did not find satisfactory results with respect to neurological improvement in PJK/F, with 55.6% not showing improvement, which may be attributed to delay in surgical intervention after the occurrence of symptoms.

The current study adds to the literature by providing insights into the potential causes and recovery of neurological deficits in patients undergoing reoperation for PJK/F. The observed higher incidence of neurological deficits in patients with PJF underscores the need to minimize PJF occurrence, particularly in individuals with prior fusions who are at greater risk. Future research should involve larger cohorts with extended follow-up periods to improve the generalizability of these findings. In addition, although proximal junctional angle was not associated with neurological deficits in our cohort, future studies should investigate patient- and surgery-specific factors—such as prior spinal fusion, bone quality, or alignment modifiers—that may predispose individuals to neurologic decline following PJK/F. Moreover, examining how different surgical interventions impact the risk of neurological deficits could provide further insights into optimizing treatment strategies and help minimize its occurrence.

The current study has several limitations that must be acknowledged. The retrospective nature of the study limits the patient selection and potentially introduces bias. The single institution nature along with a small sample size limits the generalizability of the findings and, therefore, needs to be validated in demographically different settings and larger populations. In addition, during follow-up, the response rate was relatively low, which reduced the reliability of our findings. Despite the limitations, this study provides valuable insights into an understudied area and provides a foundation for further research in the field.

## Conclusion

In patients who underwent ASD surgery with a subsequent reoperation for PJK/F, approximately 32% had motor deficits and 6% had bladder incontinence at the time of PJK/F diagnosis. Immediately after surgery, 33% returned to baseline, and at last follow-up, 87% of these patients returned to their baseline neurological function. These results underscore the benefit of reoperation to significantly improve neurological outcomes in this patient population.

## References

[1] Safaee MM, Ames CP, Smith JS (2020) Epidemiology and socioeconomic trends in adult spinal deformity care. Neurosurg 87:25–32. 10.1093/neuros/nyz454 10.1093/neuros/nyz454 31620794

[2] Slobodyanyuk K, Poorman CE, Smith JS, Protopsaltis TS, Hostin R, Bess S, Mundis GM, Schwab FJ, Lafage V (2014) Clinical improvement through nonoperative treatment of adult spinal deformity: who is likely to benefit? Neurosurg Focus 36:E2. 10.3171/2014.3.FOCUS1426 24785484 10.3171/2014.3.FOCUS1426

[3] Uribe JS, Deukmedjian AR, Mummaneni PV, Fu K-MG, Mundis GM, Okonkwo DO, Kanter AS, Eastlack R, Wang MY, Anand N, Fessler RG, La Marca F, Park P, Lafage V, Deviren V, Bess S, Shaffrey CI (2014) Complications in adult spinal deformity surgery: an analysis of minimally invasive, hybrid, and open surgical techniques. Neurosurg Focus 36:E15. 10.3171/2014.3.FOCUS13534 24785480 10.3171/2014.3.FOCUS13534

[4] Kim HJ, Bridwell KH, Lenke LG, Park MS, Ahmad A, Song K-S, Piyaskulkaew C, Hershman S, Fogelson J, Mesfin A (2013) Proximal junctional kyphosis results in inferior SRS pain subscores in adult deformity patients. Spine 38:896–901. 10.1097/BRS.0b013e3182815b42 23232215 10.1097/BRS.0b013e3182815b42

[5] Kim YJ, Bridwell KH, Lenke LG, Glattes CR, Rhim S, Cheh G (2008) Proximal junctional kyphosis in adult spinal deformity after segmental posterior spinal instrumentation and fusion: minimum five-year follow-up. Spine 33:2179–2184. 10.1097/BRS.0b013e31817c0428 18794759 10.1097/BRS.0b013e31817c0428

[6] Sebaaly A, Gehrchen M, Silvestre C, Kharrat K, Bari TJ, Kreichati G, Rizkallah M, Roussouly P (2020) Mechanical complications in adult spinal deformity and the effect of restoring the spinal shapes according to the Roussouly classification: a multicentric study. Eur Spine J 29:904–913. 10.1007/s00586-019-06253-1 31875922 10.1007/s00586-019-06253-1

[7] Yagi M, King AB, Boachie-Adjei O (2012) Incidence, risk factors, and natural course of proximal junctional kyphosis: surgical outcomes review of adult idiopathic scoliosis. minimum 5 years of follow-up. Spine 37:1479. 10.1097/BRS.0b013e31824e4888 22357097 10.1097/BRS.0b013e31824e4888

[8] Kim HJ, Iyer S (2016) Proximal junctional kyphosis. JAAOS - J Am Acad Orthopaed Surg 24:318. 10.5435/JAAOS-D-14-00393 10.5435/JAAOS-D-14-00393 26982965

[9] Kuo CC, Soliman MAR, Aguirre AO, Ruggiero N, Kruk M, Khan A, Ghannam MM, Almeida ND, Jowdy PK, Smolar DE, Pollina J, Mullin JP (2023) Vertebral bone quality score independently predicts proximal junctional kyphosis and/or failure after adult spinal deformity surgery. Neurosurgery 92:945–954. 10.1227/neu.0000000000002291 36700747 10.1227/neu.0000000000002291

[10] Lee B-J, Bae SS, Choi HY, Park JH, Hyun S-J, Jo DJ, Cho Y, Korean Spinal Deformity Society (KSDS) (2023) Proximal junctional kyphosis or failure after adult spinal deformity surgery - review of risk factors and its prevention. Neurospine 20:863–875. 10.14245/ns.2346476.238 37798982 10.14245/ns.2346476.238 PMC10562224

[11] Lopez Poncelas M, La Barbera L, Rawlinson J, Crandall D, Aubin C-E (2023) Proximal junctional failure after surgical instrumentation in adult spinal deformity: biomechanical assessment of proximal instrumentation stiffness. Spine Deform 11:59–69. 10.1007/s43390-022-00574-w 36083461 10.1007/s43390-022-00574-w

[12] Yagi M, Yamanouchi K, Fujita N, Funao H, Ebata S (2023) Proximal junctional failure in adult spinal deformity surgery: an in-depth review. Neurospine 20:876–889. 10.14245/ns.2346566.283 37798983 10.14245/ns.2346566.283 PMC10562237

[13] Hart R, McCarthy I, OʼBrien M, Bess S, Line B, Adjei OB, Burton D, Gupta M, Ames C, Deviren V, Kebaish K, Shaffrey C, Wood K, Hostin R, International Spine Study Group (2013) Identification of decision criteria for revision surgery among patients with proximal junctional failure after surgical treatment of spinal deformity. Spine 38:E1223–E1227. 10.1097/BRS.0b013e31829fedde 23778370 10.1097/BRS.0b013e31829fedde

[14] Hostin R, McCarthy I, O’Brien M, Bess S, Line B, Boachie-Adjei O, Burton D, Gupta M, Ames C, Deviren V, Kebaish K, Shaffrey C, Wood K, Hart R, Group ISS (2013) Incidence, mode, and location of acute proximal junctional failures after surgical treatment of adult spinal deformity. Spine 38:1008. 10.1097/BRS.0b013e318271319c 22986834 10.1097/BRS.0b013e318271319c

[15] Kim HJ, Bridwell KH, Lenke LG, Park MS, Song KS, Piyaskulkaew C, Chuntarapas T (2014) Patients with proximal junctional kyphosis requiring revision surgery have higher postoperative lumbar lordosis and larger sagittal balance corrections. Spine 39:E576–E580. 10.1097/BRS.0000000000000246 24480958 10.1097/BRS.0000000000000246

[16] Smith MW, Annis P, Lawrence BD, Daubs MD, Brodke DS (2015) Acute proximal junctional failure in patients with preoperative sagittal imbalance. Spine J 15:2142–2148. 10.1016/j.spinee.2015.05.028 26008678 10.1016/j.spinee.2015.05.028

[17] Ha K-Y, Kim E-H, Kim Y-H, Jang H-D, Park H-Y, Cho C-H, Cho R-K, Kim S-I (2021) Surgical outcomes for late neurological deficits after long segment instrumentation for degenerative adult spinal deformity. J Neurosurg Spine 35:340–346. 10.3171/2020.12.SPINE20604 34243161 10.3171/2020.12.SPINE20604

[18] Park S-J, Park J-S, Nam Y, Choi Y-T, Lee C-S (2021) Who will require revision surgery among neurologically intact patients with proximal junctional failure after surgical correction of adult spinal deformity? Spine 46:520–529. 10.1097/BRS.0000000000003850 33290367 10.1097/BRS.0000000000003850

[19] Yagi M, Rahm M, Gaines R, Maziad A, Ross T, Kim HJ, Kebaish K, Boachie-Adjei O, Complex Spine Study Group (2014) Characterization and surgical outcomes of proximal junctional failure in surgically treated patients with adult spinal deformity. Spine (Phila Pa 1976) 39:E607-614. 10.1097/BRS.0000000000000266 24525992 10.1097/BRS.0000000000000266

[20] Glattes RC, Bridwell KH, Lenke LG, Kim YJ, Rinella A, Edwards C (2005) Proximal junctional kyphosis in adult spinal deformity following long instrumented posterior spinal fusion: incidence, outcomes, and risk factor analysis. Spine (Phila Pa 1976) 30:1643–1649. 10.1097/01.brs.0000169451.76359.49 16025035 10.1097/01.brs.0000169451.76359.49

[21] Nguyen N-LM, Kong CY, Hart RA (2016) Proximal junctional kyphosis and failure—diagnosis, prevention, and treatment. Curr Rev Musculoskelet Med 9:299–308. 10.1007/s12178-016-9353-8 27278530 10.1007/s12178-016-9353-8 PMC4958385

[22] Rupp R, Biering-Sørensen F, Burns SP, Graves DE, Guest J, Jones L, Read MS, Rodriguez GM, Schuld C, Tansey-MD KE, Walden K, Kirshblum S (2019) International standards for neurological classification of spinal cord injury: Revised 10.46292/sci2702-1 PMC8152171 34108832

[23] Kleyweg RP, Van Der Meché FGA, Schmitz PIM (1991) Interobserver agreement in the assessment of muscle strength and functional abilities in Guillain-Barré syndrome. Muscle Nerve 14:1103–1109. 10.1002/mus.880141111 1745285 10.1002/mus.880141111

[24] Parry SM, Berney S, Granger CL, Dunlop DL, Murphy L, El-Ansary D, Koopman R, Denehy L (2015) A new two-tier strength assessment approach to the diagnosis of weakness in intensive care: an observational study. Crit Care 19:52. 10.1186/s13054-015-0780-5 25882719 10.1186/s13054-015-0780-5 PMC4344764

[25] Vanhoutte EK, Faber CG, Van Nes SI, Jacobs BC, Van Doorn PA, Van Koningsveld R, Cornblath DR, Van Der Kooi AJ, Cats EA, Van Den Berg LH, Notermans NC, Van Der Pol WL, Hermans MCE, Van Der Beek NAME, Gorson KC, Eurelings M, Engelsman J, Boot H, Meijer RJ, Lauria G, Tennant A, Merkies ISJ (2012) Modifying the Medical Research Council grading system through Rasch analyses. Brain 135:1639–1649. 10.1093/brain/awr318 22189568 10.1093/brain/awr318 PMC3338921

[26] Hyun S-J, Lee BH, Park J-H, Kim K-J, Jahng T-A, Kim H-J (2017) Proximal junctional kyphosis and proximal junctional failure following adult spinal deformity surgery. Korean J Spine 14:126–132. 10.14245/kjs.2017.14.4.126 29301171 10.14245/kjs.2017.14.4.126 PMC5769937

[27] Han X, Ren J (2022) Risk factors for proximal junctional kyphosis in adult spinal deformity after correction surgery: a systematic review and meta-analysis. Acta Orthop Traumatol Turc 56:158–165. 10.5152/j.aott.2022.21255 35703502 10.5152/j.aott.2022.21255 PMC9612636

[28] Park S-J, Lee C-S, Park J-S, Jeon C-Y, Ma C-H, Shin TS (2023) Risk factors for radiographic progression of proximal junctional fracture in patients undergoing surgical treatment for adult spinal deformity. J Neurosurg Spine. 10.3171/2023.7.SPINE23103 37657113 10.3171/2023.7.SPINE23103

[29] Hills J, Mundis GM, Klineberg EO, Smith JS, Line B, Gum JL, Protopsaltis TS, Hamilton DK, Soroceanu A, Eastlack R, Nunley P, Kebaish KM, Lenke LG, Hostin RA, Gupta MC, Kim HJ, Ames CP, Burton DC, Shaffrey CI, Schwab FJ, Lafage V, Lafage R, Bess S, Kelly MP, International Spine Study Group (2024) The T4-L1-Hip axis: sagittal spinal realignment targets in long-construct adult spinal deformity surgery: early impact. J Bone Joint Surg Am 106:e48. 10.2106/JBJS.23.00372 39292767 10.2106/JBJS.23.00372

[30] Vital J-M, Boissière L, Bourghli A, Castelain J-E, Challier V, Obeid I (2015) Osteotomies through a fusion mass in the lumbar spine. Eur Spine J 24(1):S107-111. 10.1007/s00586-014-3657-4 25416167 10.1007/s00586-014-3657-4

[31] Passias PG, Ahmad W, Williamson TK, Lebovic J, Kebaish K, Lafage R, Lafage V, Line B, Schoenfeld AJ, Diebo BG, Klineberg EO, Kim HJ, Ames CP, Daniels AH, Smith JS, Shaffrey CI, Burton DC, Hart RA, Bess S, Schwab FJ, Gupta MC, International Spine Study Group (2024) Efficacy of varying surgical approaches on achieving optimal alignment in adult spinal deformity surgery. Spine (Phila Pa 1976) 49:22–28. 10.1097/BRS.0000000000004784 37493057 10.1097/BRS.0000000000004784

[32] O’Leary PT, Bridwell KH, Lenke LG, Good CR, Pichelmann MA, Buchowski JM, Kim YJ, Flynn J (2009) Risk factors and outcomes for catastrophic failures at the top of long pedicle screw constructs: a matched cohort analysis performed at a single center. Spine (Phila Pa 1976) 34:2134–2139. 10.1097/BRS.0b013e3181b2e17e 19713876 10.1097/BRS.0b013e3181b2e17e

